# Cell Penetrating Peptides as Molecular Carriers for Anti-Cancer Agents

**DOI:** 10.3390/molecules23020295

**Published:** 2018-01-31

**Authors:** Antonella Borrelli, Anna Lucia Tornesello, Maria Lina Tornesello, Franco M. Buonaguro

**Affiliations:** Molecular Biology and Viral Oncology Unit, Istituto Nazionale Tumori IRCCS Fondazione Pascale, 80131 Naples, Italy; a.borrelli@istitutotumori.na.it (A.B.); a.tornesello@istitutotumori.na.it (A.L.T.); m.tornesello@istitutotumori.na.it (M.L.T.)

**Keywords:** peptides, chemical modifications, cell penetrating peptides (CPPs), peptide cyclization, d-amino acids, chemotherapeutic drugs, gene delivery, cellular uptake, transfection

## Abstract

Cell membranes with their selective permeability play important functions in the tight control of molecular exchanges between the cytosol and the extracellular environment as the intracellular membranes do within the internal compartments. For this reason the plasma membranes often represent a challenging obstacle to the intracellular delivery of many anti-cancer molecules. The active transport of drugs through such barrier often requires specific carriers able to cross the lipid bilayer. Cell penetrating peptides (CPPs) are generally 5–30 amino acids long which, for their ability to cross cell membranes, are widely used to deliver proteins, plasmid DNA, RNA, oligonucleotides, liposomes and anti-cancer drugs inside the cells. In this review, we describe the several types of CPPs, the chemical modifications to improve their cellular uptake, the different mechanisms to cross cell membranes and their biological properties upon conjugation with specific molecules. Special emphasis has been given to those with promising application in cancer therapy.

## 1. Introduction

Cell penetrating peptides (CPPs), formerly defined as protein transduction domains, are a large class of short amino acid sequences (5–30 residues) able to traverse biological membranes and to deliver numerous compounds including small molecules, nucleic acids, proteins, viruses, imaging agents and drugs inside the cells [1,2].

The discovery of the first protein crossing the cell membrane was made independently by two research groups in 1988 [3,4]. They observed that the human immunodeficiency virus 1 (HIV 1) trans-activating (Tat) protein was able to enter tissue-cultured cells, to translocate into the nucleus and to transactivate the viral gene expression. The α-helical domain of Tat protein spanning the residues 48 to 60, mainly composed of basic amino acids, was found as the main determinant for cell internalization and nucleus translocation [5,6]. The Tat dodecapeptide GRKKRRQRRRPQ has been shown to be the minimal functional molecule and many CPPs have been derived from the original sequence [7]. In 1991, the homeodomain encoded by Antennapedia gene of *Drosophila melanogaster* was shown to cross the neuronal membrane, to translocate into the nucleus and to cause morphological differentiation of neurons [8]. The 16 amino acid peptide (RQIKIWFQNRRMKWKK) of the third helix of the Antennapedia homeodomain, namely Penetratin peptide, was able to efficiently cross the cell membranes with an energy-independent mechanism [9].

In almost 30 years an impressive number of CPPs have been used in basic research and in preclinical studies for the treatment of several diseases such as infections, inflammation, neurodegenerative disorders and cancer [10]. However, few molecules have been evaluated in clinical trials due to the limited permeability of plasma membranes, low delivery efficiency and poor specificity for target tumor cells. Several experimental approaches have been used to produce active molecules able to specifically reach cancer cells and deliver their cargo of anticancer drugs inside the cells. The advantages of peptide-based strategies over other delivery system are multiple. Peptides are non-immunogenic molecules, usually not cytotoxic, stable in physiological conditions and effective for rapid delivery into cells of many cargoes, such as protein, other peptides or nucleic acids [11].

This review describes the advances in the development and use of CPPs mainly as carriers for anticancer therapeutics. In particular, we summarize the chemical properties, the mechanisms of cell uptake, the different molecules transported by CPPs and the ongoing clinical trials evaluating CPP-based therapeutics.

## 2. Chemical Properties of Cell Penetrating Peptides

According to their origin, CPPs are classified as: (1) protein-derived CPPs, including the Tat protein and Penetratin; (2) chimeric CPPs, such the Transportan derived from the binding of the neuropeptide galanin N-terminus to the Mastoparan toxin; and (3) synthetic CPPs, comprising oligoarginines and numerous peptide nucleic acids (PNAs) formed by synthetic nucleic acid analogues bound to pseudopeptide backbone [12,13].

Currently, the public CPPsite 2.0 database (http://crdd.osdd.net/raghava/cppsite/) contains approximately 1700 different experimentally validated peptides [14].The majority of them are linear sequences (94.5%) dominantly composed of l-amino acids (84.3%), and mostly produced through chemical synthesis (54.8%) [14].

**Figure 1:**
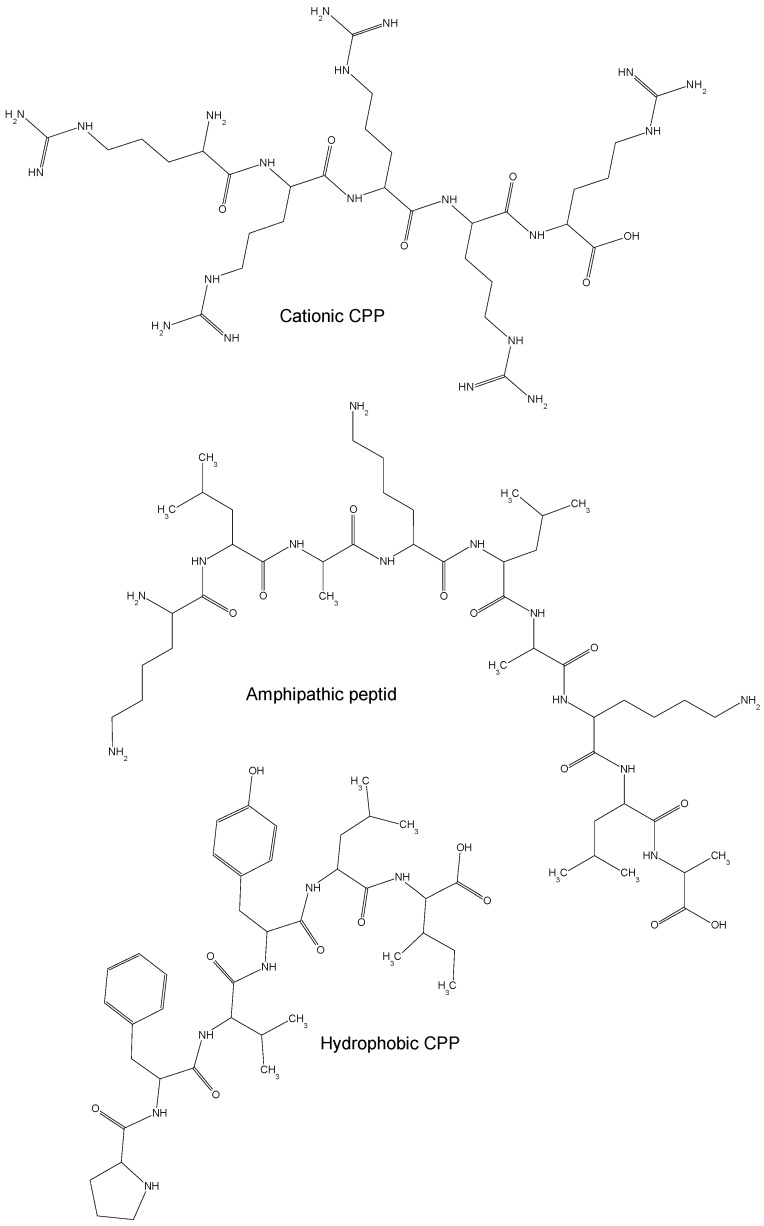
Representative structures of the three classes of CPPs: polyarginine (as cationic prototype, RRRRR), amphipathic (i.e., KLAKLAKLA) and hydrophobic (i.e., PFVYLI).

On the basis of their physical and chemical properties, CPPs (as shown in Figure 1) are classified into cationic, amphipathic and hydrophobic CPPs [15]. The majority of cationic CPPs derives from Tat and Penetratin natural peptides and usually contains more than five positively charged amino acids [16]. The poly-arginine stretches show the highest level of cell uptake thus offering higher potential for therapeutics [17]. In fact, guanidine groups in arginines form bidentate hydrogen bonds with the negatively charged carboxylic, sulfate, and phosphate groups of cell membrane proteins, mucopolysaccharides and phospholipids, respectively, leading to cellular internalization of peptides under physiological conditions [18]. Beside arginine other amino acids have shown to efficiently mediate the translocation of cationic CPPs through cell membranes. The addition of four tryptophan residues in the middle or evenly distributed along the Tat derived CPP sequence has shown to increase its cellular internalization [19]. The removal of tryptophan, instead, as for the mutated Penetratin W48F, in which residue Trp48 is substituted by a Phe, and Penetratin W48F/W56F, in which both Trp48 and Trp56 are replaced by Phe, exhibit a reduced and complete lack of cellular uptake, respectively [20]. Tryptophan has shown to be crucial for the interaction of CPPs with the phospholipid bilayer of plasma membrane [21].

The nuclear localization sequences (NLS) are a special group of short cationic CPPs containing polylysine, polyarginine or polyproline motifs which translocate to the nucleus through a multimeric complex containing 50–100 different proteins forming the nuclear pore [22]. The classical nuclear localization sequences are conventionally defined as having one (monopartite) or two clusters (bipartite) of basic amino acids separated by a 9–12 amino acid linker [23]. The simian virus 40 (SV40) T-antigen NLS (PKKKRKV) is considered the canonical monopartite sequence [24], while the nucleoplasmin is a bipartite CPP with the minimal sequence KRPAATKKAGQAKKKL [23]. Other examples of nuclear localization sequences include NF-Kb (VQRKRQKLMP), TFIIE-beta (SKKKKTKV), Oct-6 (GRKRKKRT), HATF-3 (ERKKRRRE), and SDC3 (FKKFRKF) [13]. 

The amphipathic CPPs contain both polar and nonpolar amino acidic regions, are mainly involved in the intracellular transport and accumulate preferentially in the nucleus. They include multiple antigen peptide (MAP), integrin receptor targeting peptide arginine-glycine-aspartic (RGD), and herpes simplex virus protein VP22.

Several chimeric amphipathic CPPs have been obtained by a covalent bound of a hydrophobic domain to a NLS for efficient transport through the cell membranes [15]. For instance, the primary sequence of the two chimeric peptides MPG (GLAFLGFLGAAGSTMGAWSQPKKKRKV) and Pep1 (KETWWETWWTEWSQPKKRKV) is based on the SV40 NLS (PKKRKV) linked to the HIV glycoprotein 41 (GALFLGFLGAAGSTMGA) and the tryptophan-rich cluster (KETWWETWWTEW), respectively, through the linker domain WSQP. The MPG and Pep-1 hydrophobic moieties are required for efficient targeting of the cell membrane and for forming hydrophobic interactions with proteins [15,25].

Structural studies showed that the same amphipathic peptide can adopt different secondary structures and their affinity for the hydrophobic/hydrophilic interfaces varies according to the experimental conditions [26]. Several CPPs, including Penetratin and Arg9, exhibited no affinity for the air–water interface but they have shown to interact with negatively charged phospholipids. In contrast, TP10 (AGYLLGKINLKALAALAKKIL), MAP (KLALKLALKALKAALKLA), EB1 (LIRLWSHLIHIWFQNRRLKWKKK) and M918 (MVTVLFRRLRIRRACGPPRVRV), which are the most amphipathic peptides, have shown to strongly interact with dioleoylphosphatidylcholine (DOPG). Among these, the “helix” group TP10, MAP and EB1 were shown to insert spontaneously into DOPC lipid monolayer. Moreover, the structural analysis of the peptide/lipid interactions indicated that peptides adopting a β-structure, such as M918 and MPG, were more sensitive to charges than the helix group [26]. 

The β-sheet structures are formed either by hydrophobic or by hydrophilic amino acids and are essential for cellular uptake. In fact, the substitution of l-amino acids with d-amino acids generally determines poor uptake due to the low propensity of d-amino acids to adopt the β-sheet conformation [27]. Proline-rich peptides are a class of CPPs presenting with two secondary structures: the polyproline I (PPI) and the polyproline II (PPII) [28]. PPI is the preferential conformation in the presence of aliphatic alcohols and forms a right-handed compacted helix of 3.3 residues per turn, whereas PPII is the main conformation in aqueous media or in the presence of aliphatic acids and forms a left-handed extended helix of 3.0 residues per turn [28].

Hydrophobic CPPs dominantly contain nonpolar amino acids with high affinity for the hydrophobic domain of cell membranes and can translocate across lipidic membranes in an energy-independent manner [29]. Several natural hydrophobic CPPs have been identified including the C105Y (CSIPPEVKFNKPFVYLI) peptide and its C-terminal domain PFVYLI as well as the Pep-7 peptide (SDLWEMMMVSLACQY) [30,31]. Different strategies have been used to produce chemically modified hydrophobic, such as peptide stapling for structurally-stabilized α-helical peptide with the capacity to resist proteolysis [32], prenylation to increase the affinity to cell membranes [33], and the pepducin technology to potentially identify new drug targets and to modulate the interaction of peptides with G protein-coupled receptors [34].

### Chemical Modifications of CPPs to Enhance Therapeutic Delivery

**Figure 2:**
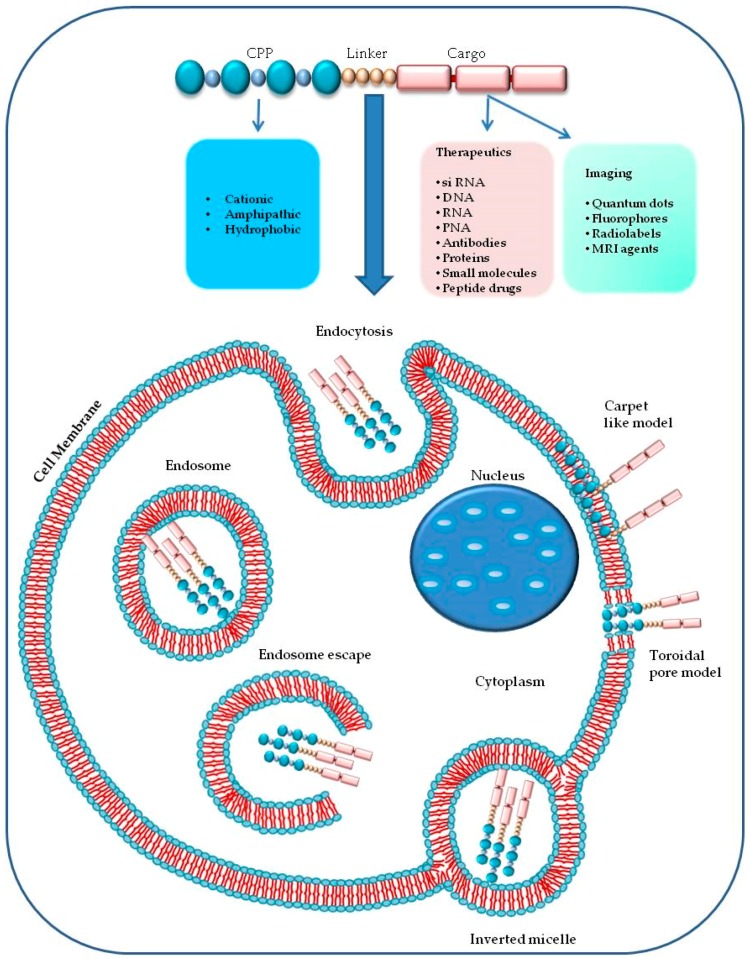
Schematic representation of several cargo types delivered by CPPs and of cellular uptake mechanisms including endocytosis (clathrin mediated endocytosis, caveolae mediated endocytosis, clathrin/caveolae indipendent) and direct traslocation (carpet like model, toroidal pore model).

A number of synthetically CPPs have been generated with chemical modifications improving cellular uptake and providing cellular and sub-cellular specific targeting (Table 1). Recent approaches include the use of low-molecular-weight prodrugs, liposomes, micro- and nanoparticles, unnatural aminoacids (Figure 2). For example, the replacement of lysines with ornithine residues confers to peptides resistance to cellular degradation [35]. Cargo delivery efficiency has also been improved by modifying the structure of peptides into dendrimers or cyclization [36,37,38]. The addition of a phosphorylated group and the addition of hydrophobic stearyl-moiety to the amphipathic CPP Trasportan 10 (TP10), improves the pharmacokinetics and the stability in the bloodstream [39].

The introduction of d-amino acids instead of their l-amino acid configuration in CPP sequences represents a common strategy to protect peptides from degradation. Several CPPs containing d-amino acids have been synthesized such as TAT, R9, penetratin, hLF, pVEC, and sweet arrow [28,40]. In addition, the extended in vivo half-lives of d-peptides over l-peptides have contributed to the successful development of d-polyarginine CPPs as cancer contrast agents [41,42].

More recently, CPPs have been used to create multifunction drug delivery systems and to specifically deliver in the cell compartments many different cargoes, including nanoparticles, proteins, liposomes and nucleic acids [43,44]. The peptide gH625 (HGLASTLTRWAHYNALIRAF) was previously identified as a membrane-perturbing domain in the glycoprotein H (gH) of the herpes simplex virus type 1; gH625 interacts with model membranes, contributing to their merging and is able to traverse the membrane bilayer and transport a cargo into the cytoplasm and across the blood–brain barrier. In particular, the gH625 peptide has been shown to transport quantum dots inside the cytoplasm in an efficient way and only partially involving endocytic pathways [45].

In a recent study, the gH625 peptide (Ac-HGLASTLTRWAHYNALIRAF-CONH_2_), was modified and conjugated to multifunctional nanoparticles composed of superparamagnetic iron oxide nanoparticle (SPION) core, cyanine fluorescent dye emitting in far red and polyethylene glycol (PEG_5000_) coating [46]. A cysteine at C-terminus was added to conjugate the CPP (Ac-HGLASTLTRWAHYNALIRAFC-CONH_2_) to the fluorescently labelled PEG shell (SPIONs-PEG-CPP). The novel nanoprobe is a multimodal imaging agent able to enter cancer cells by endocytosis and to emit far-red fluorescence allowing to detect cancer cells both via optical detection or MRI. The in vitro evaluation on the human mammary carcinoma cell line MDA-MB-231 showed that after a short incubation, SPIONs-PEG-CPP uptake was 3-fold higher than that of SPIONs-PEG. The CPP also drives the subcellular distribution of a higher nanoprobe fraction towards low polarity cytosolic locations [46]. 

A fusion peptide, has been obtained by the conjugation of Tat peptide with a nuclear localization signal protein which was able to suppress breast tumorigenesis through the inhibition of β-catenin/LEF-1 signaling [86]. More recently, a histidine-rich CPP, namely (TH) AGYLLGHINLHHLAHL(Aib)HHIL-NH_2_ (Table 1), with acid-activated pH response has been synthesized [85]. The protonation of the acid-activated CPP in a weakly acidic environment has been facilitated by the introduction of alkylated histidine analogues in the peptide sequence. In addition, the binding of methyl, ethyl, isopropyl and butyl groups to the l-histidine imidazole produced moieties suitable as pH-sensitive vectors for targeted antitumor drug delivery with low toxicity [85]. A new CPP, the arginine-rich protamine (Pro) (PRRRRSSSRPVRRRRRPRVSRRRRRRGGRRRR), containing a membrane-translocation domain fused to a nuclear-localizing sequence has been synthesized and successively used for photodynamic therapy, which together with chemotherapy and surgery for cancer treatment, cause demolition of cancer tissues with visible light in the presence of a photosensitizer and oxygen [87]. In particular, the rhodamine (Rho) has been conjugated to Pro to form RhoPro which has dual properties of membrane-internalization for the arginine-rich content on Rho and of rapid photodynamic cell death induction for the light-induced cell membrane rupture [87].

## 3. Mechanisms of Cell Uptake

The first contact between the CPPs (specifically cationic CPPs) and the cell surface occurs through electrostatic binding to proteoglycans named glycosaminoglycans (GAG) such as heparin sulfate (HS), heparin, and chondroitin sulfate B [88,89].

These glycoproteins, containing many negative charges, are ubiquitous in cell membranes [90] and constitute a platform that connects the CPPs or CPP/cargo conjugates to the extracellular matrix. The binding to GAG is followed by a selective activation of some small GTPase (RhoA and Rac 1), a remodelling of the actin network that increases the membrane fluidity [91,92] and the formation of lamellipodia [93,94,95].

The physicochemical properties of the peptide and cargo as well as other experimental and environmental factors influence the CPP interaction with the cell membrane [96]. Cationic and amphipathic CPPs show a disorganized structure in aqueous solution, but only amphipathic CPPs assume an alpha-helical structure or rarely beta-sheet structure interacting with the lipid bilayer containing hydrophilic and hydrophobic regions [97,98]. The secondary structure of amphipathic peptides is strongly correlated with the mechanism of cellular uptake [1,99,100,101]. The presence of arginines in CPPs is important because they can create hydrogen bonds with polar lipid groups [102,103] and the number and the location of arginines in the CPP sequence have been associated with a better uptake efficiency [104,105]. Substitution or deletion of arginines can decrease this uptake: an example is TAT CPP uptake [106]. Wender et al. prepared several analogues of Tat49–57 and evaluated their cellular uptake into Jurkat cells by flow cytometry. All truncated and alanine-substituted analogues showed lower uptake than Tat49–57 confirming the primary role of Tat49–57 cationic residues in the absorption. Also, they showed that l-arginine (R5–R9) and d-arginine (r5–r9) oligomers conferred a significant enhancement in the uptake compared to Tat49–57. The R9 and r9 were 20-fold and >100-fold, respectively, more efficient than Tat49–57 in the cell uptake [40]. Their studies proved that the guanidinium groups of Tat49–57 play a greater role in the internalization than either charge or backbone structure and synthesized a class of polyguanidine peptoid derivatives containing a guanidinium dominated surface [40,49].

Polylysines show a lower rate of cell uptake than polyarginines (Rx) although they are both cationic amino acids [1], arginines have a higher degree of positive charges that facilitate bidentate binding with negatively charged GAGs [95,107].

The chirality of amino acids is involved in the cell binding and uptake. Indeed l-aminoacids peptides enter more efficiently than d-aminoacids peptides even though they show the same affinity to heparan sulfate [108]. However, it is essential to note that some studies demonstrated that the inclusion of d-amino acids improves the stability of CPP for their reduced sensitivity to enzyme degradation than L forms [2,108,109]. The pathways involved in the peptide uptake have not yet been fully clarified [49].

A single CPP (as shown in Figure 2) can use different mechanisms to enter into the cell [2], and the experimental conditions such as the tumor microenvironment, the cell type, the CPP and cargo concentrations have a significant role in cellular uptake [107,110,111]. The two principal mechanisms that differ in their use of energy are (1) direct penetration and (2) endocytosis. The former is an energy-independent passive transport; the second is an energy-dependent mechanism [96].

### 3.1. Direct Penetration

The direct translocation of CPPs across the lipid bilayers occurs also at low temperatures, it is an energy-independent uptake mechanism, without the participation of receptors [40,112,113]. Three different models have been proposed to explain the CPPs internalization process in cells by direct penetration. According to the barrel-stave pore model, the hydrophilic peptide regions are oriented parallel to the plane of the lipid bilayer surface. When the number of peptides is sufficient (at least three), under high pH conditions, the complex between the fatty acids of the plasma membrane and the guanidinium groups of extracellular peptides assumes a perpendicular re-orientation to the surface of the outer membrane. The contact with the lower cytosolic pH causes the formation of transient pores allowing the interaction of the pore with the polar head of the phospholipids. CPPs take up a α-helical structure in the membrane, forming the internal face of the pore, increase the phospholipid transmembrane movements (flip-flop) and can enter by their hydrophilic regions [2,109,114,115].

The “carpet-like” model [72,89,112] describes the interaction of the positive charges of α-helical cationic CPPs and negative charges of phospholipids in the outer layer of the membrane that is covered by a “carpet” [116]. CPPs remain parallel to the surface without inserting into the lipid bilayer. When CPPs concentration is critical, they rotate on themselves creating a phospholipids redirection that produces an increase in membrane fluidity and the formation of micelles and pores in it [117].

The third model, called “toroidal pore” is a “two-stage” model in which there is the transition of the peptide from an inactive state to an active form. This different state depends on the concentration of peptide: at low levels, the peptide is in an inactive state and is disposed parallel to the player. At high concentrations, the CPP is orientated perpendicularly to the bilayer and assumes an active state, penetrating the hydrophobic regions. This contact determines an irreversible membrane destabilization, releasing the CPP into cytosolic compartment [114].

These three models should be reasonable to explain the internalization of large molecules and require that the CPPs show amphipathic alpha-helix secondary structures [112]. 

Then, several studies demonstrated that all mechanisms of direct translocation could be ascribed to the artefacts caused by cell fixation suggesting a re-evaluation of the models involved in peptides cell penetration [118,119]. This artefact distribution of CPPs into the cells suggested that the principal internalization model for CPP-cargo complexes could be ascribed to another mechanism, namely the endocytic mechanism [2,119].

### 3.2. Endocytosis

Endocytic internalization of CPPs is an energy-dependent mechanism that includes different models: macropinocytosis [120], clathrin-mediated endocytosis [121] or caveolin-mediated endocytosis [122]. Macropinocytosis is a nonspecific uptake of extracellular molecules and begins with an invagination of a membrane supported by actinic cytoskeletal elements to form first a pocket and second large endocytic vesicles containing different types of cargoes. Macropinocytosis has been involved in the uptake of some polyarginine and TAT [49,106,120]. 

Instead, passive transport is suggested for CPP penetratin because there is no actin rearrangement [105]. Clathrin-mediated endocytosis is a specific uptake of extracellular molecules. Clathrin initiates the formation of a vesicle by a crystalline coat on the inner surface of the cell’s membrane. The coated vesicles lose their coat of clathrin proteins and merge to an early endosome. The early endosome is carried via microtubules from the cell periphery towards the nucleus. The macromolecules, transported into the late endosome, fuse with vesicles of the Golgi complex where lysosomal hydrolase precursors are. These enzymes are activated, and subsequently, the late endosome modifies into an active lysosome. In the lysosome, the endocytosed material is degraded [123,124].

Caveolin-mediated endocytosis is a specific uptake of extracellular molecules and begins with a flask-shaped pit in the membrane that resembles the shape of a cave. This kind of endocytosis involves many complex events. At the first moment, the CPP with its cargo is tied to the membrane, trapped in caveolae to which are bound actinic cytoskeletal elements. This binding evokes a protein tyrosine phosphorylation and then a depolymerization of actin elements. An actin patch is formed, and dynamin (a GTPase responsible for endocytosis in the eukaryotic cell) determines another actin polymerization on the patch. The cargo-loaded vesicles are released into the cytosol [125,126]. There is evidence that the endocytic uptake pathways mediate intracellular delivery of high molecular weight cargos or large proteins [127].

### 3.3. Escape from Endosomes

During endocytosis, CPPs, with their cargo, could be entrapped into endosomes or lysosomes [2,10] and be degraded without having the possibility to reach their target sites (nucleus, mitochondrion) to exert their biological activity. The delivery of macromolecules and nanoparticles can occur via an endocytic mechanism, but it is essential to escape from endosomes [2,128]. Several models suggest the way CPPs could overcome the endosomes that are the principal limiting factors for the right delivery of CPP-cargo complexes. According to one mechanism, the interaction between the negative charges of the endosomal membrane and the positively charged components of CPPs occurs. This contact causes a membrane stiffening and rupture determining the release of the vesicle’s contents [129].

Another example suggests the importance of the pH gradient change; a reduced pH increases the bonding ability of CPPs to the endosomal membrane and afterwards, their intracellular delivery. The increase of the endocytic vesicles might improve the escape from endosomes [2]. 

These observations can be considered decisive to improve the efficiency of the endosomal escape of macromolecules (nucleic acids) noncovalent complexed with amphipathic CPPs. However, there is no evidence that these models might apply to cationic CPPs covalently bound to large cargoes that are more easily trapped in endosomes [128,130].

### 3.4. Chimeric and Synthetic CPPs

Several peptides promote the escape from endosomes. Some of them are short peptides derived from the influenza virus protein hemagglutinin (INF) [131,132], while others, such as GALA, KALA and melittin, are synthetically designed peptides [49,133,134].

GALA peptides allow the fusion to the endosomal membrane, and release of cargoes into the cytosol, increasing the transfection efficiency [135]. Salomone et al. described a novel chimeric peptide containing the TAT 11 motif bound to the CM18 hybrid (KWKLFKKIGAVLKVLTTG), residues 1–7 of Cecropin-A and 2–12 of Melittin), which has two important functionalities: efficient uptake and destabilization of vesicle membranes. This fusion allows increasing cargo-molecule cytoplasm translocation and intracellular localization of several membrane impermeable molecules such as plasmid DNA and calcein [136].

Various other synthetic agents improved the ability of endosomal escape of CPPs, some of which are the PepFects (PF5 and PF6) derived from the amphipathic CPP Transportan 10 (TP10). The stearyl-TP10 has been modified by adding an N-terminal sterylation that increases its endosomal escape [137]. PF6 was obtained by incorporating the lysosomotropic agent chloroquine in stearyl-TP10 to promote the escape, while PF15 was obtained by replacing the lysine residues with ornithines to improve the capacity for endosomal escape [138].

## 4. CPPs and Anti-Cancer Drug Delivery

Cancer is a major cause of death worldwide and chemotherapy is the most common therapeutic approach [139]. However, major problems of chemotherapeutics are the poor penetration of drugs into tumor tissues, the appearance of resistant tumors to high dosage or to long-term treatment and dose-dependent side effects. The low penetration of drugs in the cancer tissues is due to a dense connective stroma that hinders the entry of molecules into tumoral tissue (pancreatic cancer is an example) and the presence of high interstitial pressure caused by abnormal blood and lymphatic vessels. CPPs can improve the drug delivery in tumor cells by facilitating extravasation and penetration of cancer cells while other tissues remains unaffected by the drug. Therapeutic strategies that use antibodies or peptides recognizing target molecules specific to tumor cells allow to concentrate into tumoral tissue an amount of drug able to inhibit cancer growth. Thus, increased activity and reduced toxicity to healthy tissues are expected when the drug is localized and accumulated preferentially in the tumor site [140]. Endogenous stimuli such as activation of specific enzymes or pH value which characterize different cancer cells can increase cell specificity. Also extrinsic stimuli, such as mild heat have had encouraging results enhancing cargo delivery and increasing the accumulation of CPP delivered drugs.

### 4.1. CPPs for Delivery of Chemotherapeutic Agents

The conjugation of some anticancer drugs such as Taxol [141], Methotrexate (MTX) L [47], Doxorubicin (Dox) to CPPs increases the membrane permeability, the drug delivery, the drug half-time circulation and the accumulation in tumor cells [142,143].

R8 CPP, linked to taxol via disulfide linkers, improves the aqueous solubility and pharmacokinetics of the drug and overcomes the multiple drug resistance (MDR) compared to the drug alone [141]. MTX, conjugated to two different CPPs, YTA2 (Acetyl-YTAIAWVKAFIRKLRK-amide) and YTA4 (Acetyl-IAWVKAFIRKLRKGPLG-amide) (Table 1) has shown to kill cancer cells more efficiently than MTX alone [47].

Many CPPs can conjugate the Dox and may improve intracellular delivery of the drug. The most used are TAT, Pen, which have reported to induce apoptosis in hamster (CHO) and human cancer cells (HUVEC, MDAMB231, MCF-7 cells) at low doses [142,144].

Another drug delivery system, capable of overcoming the MDR, was obtained by conjugation of CPPs to mesoporous silica nanoparticles surface. The TAT peptide-MSNs-Dox complex enhanced the intracellular and intranuclear Dox delivery in multidrug-resistant MCF-7/ADR breast cancer cells much more efficiently than free Dox [145]. Walker et al. conjugated three different CPPs to Dox to inhibit tumor growth in mice. One of them was an Elastin-like polypeptide (ELP), a temperature-sensitive peptide polymer, enabling a phase transition from liquid to solid (or the reverse) depending on the temperature [146]. This feature allowed to deliver and to accumulate the drug in solid tumors when treated with localized hyperthermia. The CPP–ELP–Dox compound inhibited tumor growth much more efficiently than free Dox at the same concentration [146].

The cellular uptake of several fluorescence-labelled drugs (i.e., lamivudine) was significantly increased by the presence of cyclic peptide [WR]4-AuNPs in human ovarian adenocarcinoma (SK-OV-3) cells [147]. This peptide containing tryptophan and arginine residues showed a low cytotoxicity and improved drug cellular uptake and delivery [147]. Moreover, Vincristine sulfate or Paclitaxel conjugated R7 CPP (Table 1) and TATp-modified PEG-PE micelles, respectively, showed a significant increase in the in vitro cytotoxicity to different cancer cells [50].

### 4.2. CPP and Nuclear Acids Delivery for Anti-Cancer Therapy

Many diseases such as cancers, or other genetic and nongenetic disorders could be treated by gene therapy [139,148]. The molecules for this type of treatment are large and hydrophilic, thus they are not able to cross cell membranes and need to be associated with a delivery vector. At first, viral vehicles were used with an excellent efficiency, but with high toxicity and immunogenicity [149]. Most of the nonviral vectors (liposomes, cationic polymers, etcetera) are cationic and have a low toxicity. They can create electrostatic interactions with negative charges of DNA, but they have a lower transfection efficiency compared to viral carriers. CPPs assemble oligonucleotides and plasmid DNA into nanoparticles possessing positive charges that allow them to interact with cellular membranes internalizing CPP-cargo complexes [11,150]. Liu et al. demonstrated that using arginine-rich CPPs (SR9, PR9, and HR9) (Table 1) to transfer plasmid DNA into A549 cells enhanced the gene expression levels [81]. Veiman et al. showed that PepFect14 peptide (Table 1) could deliver plasmid DNA forming stable nanoparticles that improved the transfection efficiency in cell lines and primary cells by caveolae-mediated endocytosis [82,83].

A new method associated the concept of intracellular delivery with a transposition of mobile elements such as piggyBac (PB) transposase that is a genetic element that transposes between CPP and plasmid DNA. This method is considered a good option for a potential gene therapy [151].

To improve the low transfection efficiency of TAT CPP bound to DNA, Saleh et al. attached the membrane-active peptide LK15 to TAT (RKKRRQRRRGGGKLLKLLLKLLLKLLK), increasing the gene delivery and enhancing the gene expression in cell lines [36]. Mann et al. chemically modified the cationic peptide Murine VE-cadherin (pVEC) changing its amphipathic form from primary to secondary and produced a sequence of nine arginines in the peptide adding some histidines too. This structural rearrangement strongly enhanced DNA delivery efficiency, especially in the presence of chloroquine and its analogues that are critical to enhance the endosomal escape capacity [138,152]. Two amphipathic peptides (MGP and Pep-1) have shown to improve the nuclear translocation of DNA without breaking the cell membrane during mitosis. They are composed of three parts: an N-terminal hydrophobic domain, a hydrophilic domain that can react with oligonucleotides being rich in lysines and a linker domain presenting a proline amino acid that gives greater flexibility to the other two domains [153].

Another approach to improve the delivery efficiency and the specificity of CPPs—cargo complexes is the conjugation of a CPP to a targeting ligand, for example the TAT-Mu (TM) peptide combined with HER2 antibody mimetic-affibody (AF). This conjugation designated TMAF, allows the specific transgene expression, binds DNA efficiently and protects plasmid DNA from DNase I action [154].

Regarding cancer treatment strategies based on the use of nucleic acids, siRNAs play a significant role but have a poor cellular uptake [155]. It is possible to overcome this obstacle by adding to the siRNA, oligoarginine-modified chitosan that improves the siRNA delivery into cells with low toxicity [156]. TAT peptide can also be used to deliver siRNA inside the cells and the addition of calcium dramatically increases the efficiency of transfection particularly when a longer TAT is complexed with siRNA. Calcium allows a condensation of siRNA-TAT into smaller nanoparticles and enhances the knockdown of luciferase expression with a lower levels of cytotoxicity [157]. The conjugation of TAT CPP with an esapeptide (A1) showing high affinity for VEGFR1 improves the delivery of siRNA into cells inducing a greater gene silencing [158]. The siRNA-CPP nanoparticles delivery efficiency depends on the resistance to serum protein, decomposition due to polyanions and to cellular uptake [159]. A modified amphipathic peptide MPG, named MPG-8 (AFLGWLGAWGTMGWSPKKKRK), was used to form nanoparticles with siRNA targeting cyclin B1, which is up regulated in several cancer types. The pharmacokinetic of the MPG-8/siRNA compound was optimized by functionalization with a cholesterol moiety [160]. This compound was injected intravenously in mice bearing xenografted tumors and induced a significant reduction in tumor size [160]. 

A further approach to deliver siRNA has been to use a TAT protein fused to a double-stranded RNA-binding domain (TAT–DRBD) highly specific for siRNAs [161]. The TAT–DRBD system was used for intracranial delivery of epidermal growth factor receptor (EGFR) and AKT serine/threonine kinase 2 (Akt2) siRNAs to treat glioblastoma in mouse models and it was showed to induce a lethal RNAi responses and increased animal survival [162]. Wang et al. developed an electrostatic complex between R9 CPP and siRNA silencing polo-like kinase-1 (Plk-1). This compound was delivered to MDA-MB-231 breast cancer cells in vitro and in vivo and was shown to decrease tumor growth in both cases [89,163].

### 4.3. CPP and Protein Delivery for Anti-Cancer Therapy

More recently, the new direction of translational research is focused heavily on the use of therapeutic proteins and vaccine peptides in anticancer therapies. The first response to a peptide-based vaccine is a humoral antibody response. The activation of cytotoxic T-cell lymphocytes (CTLs) requires the antigen processing via the MHC class I pathway and CPPs can foster the cytosolic uptake of vaccine-peptides through the MHC I presentation. LAH4, a non-covalently bound cell-penetrating peptide, enhanced the cellular delivery of a tyrosinase-related protein 2 (TRP2) peptide vaccine, inducing anticancer effect in tumor tissues expressing TRP2 in mouse models [131,164].

A novel amphipathic CPP, p28 (Table 1), is derived from a redox protein (azurin) from the pathogen *Pseudomonas aeruginosa*. A minimal protein domain of azurin (amino acids 50 to 67) named p18, contains the cell penetrating properties and enables azurin to enter into human cancer cells. Fragment p28 (amino acids 50 to 77), additionally, can influence p53, can bind and stabilize p53 producing p53-mediated apoptosis and antitumor effects [165]. This CPP p28 also has anti-angiogenic effects and entered clinical trials in humans [166,167].

Although many clinical trials based on the use of CPP are underway no CPP-based drug has obtained US Food and Drug Administration (FDA) approval as a cancer therapeutic [89]. Clinical applications of CPPs show several disadvantages and limitations. CPPs can be degraded by enzymes circulating in the plasma. The use of protease resistant-CPP is the first useful strategy to resolve this obstacle [108]. Sometimes, CPPs have been sterically shielded by polyethylene glycol (PEG) chains attached to the surface by a linker that can be cleaved via stimulus from tumor tissues, changing local environmental conditions and inactivating PEG protection [168,169].

### 4.4. “Smart” Intracellular Drug Delivery Systems for CPP-Mediated Cancer Therapy

A novel system to control the safety of CPP-mediated intracellular drug delivery is based on the use of a prodrug made up of two components. The first element is an antibody which is a heparin-linked tumor-targeting unit or a magnetic iron oxide nanoparticle (MION) carrier that represents a targeting component. The second element is a drug complex consisting of a covalent polycationic CPPs, such as TAT or LMWP, and a macromolecular drug, such as a protein or a nucleic acid [170,171].

The drug candidate is a protein toxin or a siRNA which is linked via a degradable S-S bond to nontoxic CPP with low molecular weight such as LMWP. The targeting unit is a MION carrier with a superparamagnetic feature and is covered with a biocompatible heparin-dextran polymer. Together constitute an LMWP-modified drug and the heparin-coated MION as unique pro-drug. The binding between LMWP and heparin of a MION carrier hinders the cell-penetrating function of LMWP during tumor targeting. When the concentration of the MION carriers in the tumor is high, the administration of protamine, a heparin antidote that binds heparin stronger than LMWP, allows the release of LMWP-drug. At the end the drug is removed from LMWP through the degradation of the S-S bond leading to the initiation of tumor death by apoptosis [172].

### 4.5. Increasing Cell Specificity Systems for CPP-Mediated Cancer Therapy and Diagnosis

The mechanism of CPP internalization based on the binding of the peptides to bilayer phospholipids is unspecific and represents a severe obstacle to the clinical use of CPPs. A promising system to increase the specificity is represented by the activatable CPPs (ACPPs) obtained by coupling shielding polyanions to the peptide with target-specific cleavable linkers. Tissue-specific proteases, such as a matrix metatalloprotease 2/9, detach the linker from the inhibitor domain enabling the cleaved ACPP to enter the cancer cells. In 2009, the group of the Nobel Prize winner Prof. Roger Y. Tsien developed the first protease-based ACPP able to cleave the linker between the polycationic CPP and his inhibitor domain enabling the peptide to deliver cargoes specifically in tumor cells [2].

Liu et al. used a legumain-cleavable linker, the alanine-aspargine-alanine (AAN) sequence, which is digested by the legumain protease, an enzyme overexpressed in several solid tumors. Liu used the AAN sequence to branch TAT which was then entrapped into liposomes to deliver Dox into the cells. The addition of AAN-TAT-liposome-Dox to legumain-expressing 4T1 cells showed accumulation and internalization of Dox. Moreover, the treatment of 4T1-orthotopic mice with AAN-TAT-liposome-Dox induced tumor regression [173].

Savariar et al. used the ACPP system as a tumor-detection method. This ACPP was based on the nona-arginine (R9), octa-glutamate (E8), labelled with Cy5 and Cy7, respectively, and the MMP2/9 cleavable linker (PLGCAG). When MMPs in the tumor tissue cleave the linker a fluorescence resonance energy transfer (FRET) between Cy7 and Cy5 were also interrupted producing red fluorescence by Cy5 in the tumor tissue [174].

Tan et al. developed another strategy to target selectivity the cancer cells by fusing an anti-Her-2/neu mimetic peptide to a Tat-derived CPP. The anti-Her-2/neu mimetic peptide recognises the epidermal growth factor ErbB2, which is overexpressed in 30% of breast cancers. The conjugation of the targeting ligand with a CPP improved the efficiency of specific uptake in treated cancer cells [89,175].

Few CPPs have been used for cancer diagnosis by molecular imaging techniques such as SPECT, PET, optical imaging, and MRI. The sensitivity of such techniques relies on the efficient delivery of contrast agents to the cytoplasm and/or nuclei of the target tissue. Thus, a key strategy to overcome this problem has been the introduction of CPPs in the design of new contrast agents. Nguyen and his collaborators developed a method to see the tumors during surgery based on activatable cell-penetrating peptides (ACPPs), in which CPP is fluorescently labelled and coupled via a cleavable linker to a neutralizing peptide. The presence of proteases specific for tumor tissue allows the cleavage of linker dissociating the inhibitory peptide from CPP that can enter into the cells. Into immunocompetent mice grafted with syngeneic cells derived either from spontaneous tumors in transgenic mice, Cy5-labeled free ACPP and ACPPD (Cy5 and gadolinium–DOTA) allowed to delineate the margin between tumor and adjacent tissue, resulting in a superior precision of tumor resection. Surgery guided by ACPPD enabled preoperative whole-body tumor detection by MRI, intraoperative guidance by real-time fluorescence, intraoperative histological analysis of margin status by fluorescence, and postoperative MRI tumor quantification [176].

### 4.6. CPP and Organelle-Specific Delivery for Anti-Cancer Therapy: Mitochondrial Delivery

Mitochondria are the powerhouses of cells, and they control several programmed cell death mechanisms. One of the hallmarks of the cancer is the hypoxic condition that is lethal for healthy cells. Under inadequate amounts of oxygen, mitochondria do not produce enough ATP, tumor cells overexpress hypoxia-inducible factor-1 (HIF-1), and the induction of this factor upregulates the glycolytic pathway. Accumulation of Krebs cycle substrates such as succinate occurs. Malignant cells are characterized by decreased expression or mutated p53 which cause the escape from hypoxia-mediated cell death. The mutated tumor suppressor protein causes a down-regulation of mitochondrial respiration an up-regulation of glycolysis. Reactive oxygen species (ROS) are produced during a normal cellular function. Also, a dysfunctional mitochondrial respiratory chain produces an abnormal amount of ROS that are extremely reactive and unstable species. This chemical reactivity induces lipid peroxidation and protein oxidation and degradation. ROS induce damage mitochondrial DNA (mtDNA) in the form of mutations, deletions, gene amplification, and rearrangements. The mitochondria-mediated intrinsic apoptotic pathway is suppressed, and the activation of oncogenes and the inactivation of suppressor genes can occur (over-expressed anti-apoptotic proteins such as Bcl-2, Bcl-XL, Mcl-1, and Bcl-w). These changes are typical of cancer cells [177,178]*.*

Since mitochondria play this critical role in mediating cell death, could be promising to have anti-cancer drugs targeting the mitochondria. Horton et al. [179] described a class of synthetic and natural peptides defined as mitochondrial-penetrating peptides (MPP) that have a cellular uptake like cationic CPPs and can deliver the cargo to mitochondria. Recently a dual role antioxidant and mitochondria-penetrating peptide, mtCPP1, was prepared from the Szeto-Schiller (SS) tetrapeptide antioxidants that enter the cell by an independent energy mechanism and localizes to the inner mitochondrial membrane. mtCPP-1 was able to deliver 5(6)-carboxyfluorescein (5-FAM) across the cell membrane and preferentially target it to the mitochondria. It is also able to decrease Ros production [131,180,181].

## 5. Conclusions

The discovery of small peptides able to cross cell membranes led to the development of new drug delivery systems. The numerous studies obtained from multiple preclinical and clinical trials have clearly demonstrated the ability of CPPs to increase the therapeutic response in different types of human disease including cancer.

In the last decade several CPP-based drugs have been evaluated for their activities in phase I/II clinical trials. The AZX100, a phosphorylated peptide analogue of heat shock protein 20 (HSP20) fused to a Tat peptide derived sequence (PTD4, YARAAARQARA) has shown to reduce keloids, surgical scarring and fibrotic disorders (NCT00811577, NCT00825916). The RT001, made of botulinum toxin type A molecule fused to a positively charged lysine-rich central peptidic domain situated between two Tat peptides, for the treatment of lateral canthal lines, crow’s feet, and facial wrinkles (NCT01124565). The KAI-9803, the δ-PKC-selective inhibitor peptide dV1-1 conjugated to Tat peptide, has been found effective to limit tissue damage following myocardial infarction (NCT00093197).

Few clinical trials evaluated the efficacy of CPP-based drugs for cancer therapy. The p28, a 28-amino-acid peptide derived from the bacterial protein azurin, has shown to cross the cell membrane and to enter the nucleus where it suppresses p53 degradation causing inhibition of the cell cycle and cancer cell proliferation [182]. The p28 entered a Phase I clinical trial for treatment of p53-positive progressive central nervous system tumors resistant to standard treatments (NCT00914914 and NCT01975116). The results showed that p28 was well tolerated with no immunogenicity and effective in patients with refractory disease [168].

Several cancer types are treated with the irinotecan, a prodrug that is converted into its active metabolite SN38 by the action of liver carboxylesterases. The SN38 drug cannot be administered directly due to its high insolubility, then the DTS-108, a water-soluble compound comprising SN38 linked to the highly charged oligopeptide DPV1047 (Vectocell1), has been produced and evaluated in preclinical and clinical studies [183,184]. The antitumoral efficacy of DTS-108 was dose-dependent when evaluated in lung human tumors and superior to irinotecan [185], while DTS-108, a novel peptidic pro-drug of SN38, has been used in a phase I clinical study for the treatment of advanced/metastatic solid tumors [186].

The major obstacle to CPP-based therapies has been the limited cell type specificity, given that most CPPs are internalized by all cell types, their poor stability in bloodstream and their low bioavailability in target tissues due poor tissue penetration and/or cellular uptake. Several strategies have been developed to increase CPP specificity to diseased tissues such as the conjugation of CPPs with homing peptides or with other targeting ligands such as RGD peptides, folic acid and hyaluronic acid [187]. These active ligands are usually highly expressed in several tumor types but not in normal cells thus healthy tissues remain unaffected by drug delivery.

Pharmacokinetics and short blood plasma half-life can be improved by using unnatural amino acids and by coupling CPPs and drugs to macromolecular carriers, such as liposomes or biopolymers. The absence of cell specificity has been addressed by variety of controlled delivery strategies using endogenous or extrinsic stimuli to selectively increase uptake of CCP at the disease site. 

It should be considered that the conjugation of a CPP with cargo could sometimes generate new epitopes inducing immune responses, while a single CPP did not elicit any immune responses. Further experimental and clinical studies are needed to optimize the use of CPPs for delivery of anticancer drugs.

## Figures and Tables

**Table 1:** List of CPP-derived molecules selected from CPPsite 2.0 database and from the literature for in vivo and/or in vitro anticancer therapy.

Name	Peptide Sequence *	Activity	Cells/Tumors	Cargo	Ref.
YTA2 YTA4	YTAIAWVKAFIRKLRK IAWVKAFIRKLRKGPLG	CPP conjugated to the methotrexate (MTX) as therapeutic for drug resistant tumor cells.	Breast cancer cells MDA-MB-231	MTX	[47]
TAT Pen R12 R16 r8 r12 R5 R7	GRKKRRQRRRPPQC RQIKIWFQNRRMKWKKGC RRRRRRRRRRRRGC RRRRRRRRRRRRRRRRGC RrrrrrrrGC rrrrrrrrrrrrGC Fl-ahx-RRRRR RRRRRRR	Accumulation of oligoarginine peptides in tumor-xenografted mice.	Nude mice implanted with HeLa and CHO-K1 cells	Doxorubicin Paclitaxel	[40,48,49,50]
Glu-Oct-6 Glu-Lys 6-Oct Phe-Oct-6 Asn-Oct-6 Tyr-Oct-6	EEEAAGRKRKKRT EEEAAKKK GRKRKKRT FFFAAGRKRKKRT NNNAAGRKRKKRT YYYAAGRKRKKRT	CPP with enhanced nuclear localization in prostate cancer cells.	Prostate cancer cells DU-145 and LNCaP	Nucleic acid	[51]
RV24	RRRRRRRRRGPGVTWTPQAWFQWV	Amphipathic peptide-carrier for targeting cancer cells.	T98G, HepG2 and HeLa cells	β-galactosidase and eGFP	[52]
TAT-NBD TMTP1-TAT-NBD	YGRKKRRQRRRGTALDWSWLQTE CGNVVRQGC-G-YGRK-KRRQRRR-G-TALDWSWLQTE	Anticancer effects and inhibition of tumor metastasis by the TMTP1 compound peptide.	BALB/c nu/nu mice; PC-3M-1E8, MDA-MB-231, MCF-7 and PC-3M-2B4 cells	Tumor molecular targeted peptide 1 (TMTP1)	[53]
AgNP-TAT	CGGGYGRKKRRQRRR	TAT-modified nanosilver for multidrug-resistant cancer.	Nude mice implanted with B16 melanoma cells; Caco-2 cells	Nanosilver Nanoparticles	[54]
Crotamine	YKQCHKKGGHCFPKEKICLPPSSDFGKMDCRWRWKCCKKGSG	Crotamine as carrier for anti-cancer molecules.	B16-F10, HCT116, 3T3 cells; C57BL6 or nude mice	Nucleic acid	[55]
1 (TAT) 2 7 10 28 30 33 44 45 47 48	YGRKKRPQRRR DSLKSYWYLQKFSWR KLWMRWWSPTTRRYG RLWMRWYSPWTRRWG RLIMRIYAPTTRRYG RLYMRYYSPTTRRYG RLWMRWYSPRTRAYG KRPTMRFRYTWNPMK WKCRRQCFRVLHHWN WKCRRQAFRVLHHWN WKARRQCFRVLHHWN	-TAT derived CPP as anticancer molecular delivery systems. -Co-delivery of doxorubicin and paclitaxel using multi-functional micelles.	NOD-SCID mice model of xenograft human tumor cells; Many Human neoplastic cells including HeLa, Lovo, A549, MCF-7,MKN45,HepG2, LNCap, KPK,U2OS, RC15,RDES,H28,K562,U251,NHDF KB cells	Doxorubicin and Paclitaxel	[56]
R9-GO-203	rrrrrrrrrcqcrrkn	Inhibition of the MUC1-C oncoprotein and delivery of cytotoxic agents in breast cancer cell lines.	MCF-7 and ZR-75-1 cell lines	Taxol and Doxorubicin	[57]
iRGD-CDD	CRGDKGPDC	Proapoptotic peptide to intratumorally spreading cancer therapy.	Athymic nude mice and Balb/C mice; Human embryonic kidney (HEK) 293T, prostate cancer PPC1, mouse breast cancer 4T1, Human tumor cell line M21, Human breast cancer cell line MCF-10CA1a.	Bit1 (a pro-apoptotic mitochondrial protein)	[58]
P7-4 P7-5 P7-6 P7-7 R7-KLA KLA-R7	RRRRRRRGGIYLATALAKWALKQGF IYLATALAKWALKQGFGGRRRRRRR RRRRRRRGGIYLATALAKWALKQ IYLATALAKWALKQGGRRRRRRR RRRRRRRGGKLAKLAKKLAKLAK KLAKLAKKLAKLAKGGRRRRRRR	Membrane permeabilization by peptides with anticancer properties.	Male white rats; Jurkat and CHO	Peptide P7–27	[59]
P1 P2 P3 P4	RGD-ADDA-RRRRRRRR RGD-Ahx-RRRRRRRR RGD-RRRRRRRR RRRRRRRR	Self-assembled BolA-like amphiphilic peptides as viral-mimetic gene vectors.	293T and HeLa	Plasmid DNA	[60]
MG2A	GIGKFLHSAKKFGKAFVGEIMNSGGKKWKMRRNQFWVKVQRG	Penetratin-mediated delivery for antitumor activity of the cationic antimicrobial peptide Magainin II.	HeLa and A549 cells	Antimicrobial peptide MG2A	[61]
CRGDK	CRGDK	Functionalized micelles for delivery of anticancer drugs.	Breast MDA-MB-231 and prostatic PC3 cancer cell lines	Doxorubicin	[62]
L1	CTSTTAKRKKRKLK	Lipopeptides derived from human papillomavirus type-16 capsid for gene delivery.	Malignant human glioma cells U87MG and COS-7 cells	Plasmid DNA and siRNA	[63]
oligoarginine	rrrrrrrr	Multifunctional liposomes for targeted therapy of prostate cancer.	22Rv1 xenograft murine model; PC-3 cells	Folate	[64]
GC/R8-Lip	RRRRRRRR	Octaarginine-modified liposome as carriers of alpha-galactosylceramide.	C57BL/6 (H-2b) female mice; JAWSII cells	GC (-galactosylceramide), ovalbumin	[65]
p21-ELP1-Bac Bac-ELP43 Bac-ELP63 Bac-ELP122	RRIRPRPPRLPRPRPRPLPFPRPG	Therapeutic peptide based on thermo-responsive elastin-like polypeptide.	Female athymic nude mice (Ncr-nu/nu); S2013, Mia PaCa-2 and Panc-1.	p21 peptide	[66,67]
TP10-SRC1LXXLL R7-SRC1LXXLL TP10-SRC1(1222–1245) R7-SRC1(1222–1245)	PKKKRKV-AGYLLGKINLKALAALAKKIL- PQMQQNVFQYPGAGMVPQGEANF PKKKRKV-RRRRRRR-YSQTSHKLVQLLTTAEQQ PKKKRKV-AGYLLGKINLKALAALAKKIL- PQMQQNVFQYPGAGMVPQGEANF PKKKRKV-RRRRRRR-PQMQQNVFQYPGAGMVPQGEANF	LXXLL peptide to convert transportan 10 to a potent inducer of apoptosis in breast cancer cells.	MCF-7 cells	Peptide (LXXLL)	[68]
pep5-cpp N-pep5-cpp N2-pep5-cpp N3-pep5-cpp C2-pep5-cpp C3-pep5-cpp * C4-pep5-cpp C5-pep5-cpp C6-pep5-cpp C7-pep5-cpp A B C Ac-pep5-cpp	WELVVLGKL-YGRKKRRQRRR ELVVLGKL-YGRKKRRQRRR LVVLGKL-YGRKKRRQRRR VVLGKL-YGRKKRRQRRR WELVVLG-YGRKKRRQRRR WELVVL-YGRKKRRQRRR WELVV-YGRKKRRQRRR WELV-YGRKKRRQRRR WEL-YGRKKRRQRRR WE-YGRKKRRQRRR WELVVA-YGRKKRRQRRR WEAVVL-YGRKKRRQRRR WEAVVA-YGRKKRRQRRR Ac-WELVVL-YGRKKRRQRRR	Peptide derived from g1/s cyclin d2 that induces cell death.	C6 rat; HeLa cells	Pep-5 derivatives	[69]
C24-LMWP	VSRRRRRRGGRRRR	Low-molecular-weight protamine-modified PLGA nanoparticles for overcoming drug-resistant breast cancer.	BALB/c-nu nude mice; A549/T and MCF-7/ADR	LMWP/PLGA nanoparticles and doxorubicin	[70]
TAT-gelonin	YGRKKRRQRRR	Combination of antibody targeting and PTD-mediated intracellular toxin delivery for colorectal cancer.	C57BL/6 mice; LS174T and HCT116, MDCK and 293 HEK	Gelonin	[71]
TAT-BID	YGRKKRRQRRR	Controlled delivery of BID protein fused with TAT peptide sensitizes cancer cells to apoptosis.	PC3, LNCaP, A549, and HeLa	BID protein	[72]
PTX-TAT-LP PTX-C-TAT-LP PTX-N-TAT-LP	CAYGRKKRRQRRR CAYGRKKRRQRRR CYGRKKRRQRRR	Tumor-targeted paclitaxel delivery and enhanced penetration using TAT-decorated liposomes comprising redox-responsive poly(ethylene glycol).	B16F1 tumor-bearing C57 mice; Murine B16F1 melanoma tumor cells	Paclitaxel (PTX)	[73]
B1 B1-Leu B1-Lys	VKRFKKFFRKLKKSV VKRFKKFFRKLKKLV VKRFKKFFRKLKKKV	Design, synthesis and biological evaluation of novel peptides with anti-cancer and drug resistance-reversing activities.	MCF-7 cells	B1 peptides	[74]
TAT-LP-PTX T7/TAT-LP-PTX T7-LP	CAYGRKKRRQRRR CAYGRKKRRQRRR CHAIYPRH	Efficacy of dual-functional liposomes containing paclitaxel for treatment of lung cancer.	BALB/c male athymic nude mice; A549	Paclitaxel (PTX)	[75]
TP TP-biot1 TP-biot13 TP-10 TP10-biot1	GWTLNSAGYLLGKINLKALAALAKKIL GWTLNSAGYLLGKINLKALAALAKKIL GWTLNSAGYLLGKINLKALAALAKKIL AGYLLGKINLKALAALAKKIL AGYLLGKINLKALAALAKKIL	Protein and siRNA delivery by transportan and transportan 10 into colorectal cancer cell lines.	HT29 and HCT116	siRNA	[76]
Peptide 1 Peptide 2 Peptide 3 Peptide 4 Peptide 5 Peptide 6 Peptide 7 Peptide 8 Peptide 9 Peptide 10 R9 Peptide 1-C3G Peptide 1-NΔ Peptide 1-SΔ Peptide 1-NSΔ Peptide 1-NTSΔ Peptide 1-NTCSΔ Peptide 1-NTHSΔ	NTCTWLKYHS CASGQQGLLKLC YNNFAYSVFL ECYPKKGQDP RHVYHVLLSQ HATKSQNINF YRDRFAFQPH IWRYSLASQQ YQKQAKIMCS VQLRRRWC RRRRRRRRR NTGTWLKYHS TCTWLKYHS NTCTWLKYH TCTWLKYH CTWLKYH TWLKYH CTWLKY	Novel cell-penetrating peptide targeting human glioblastoma cell lines.	U87MG cells	p16(INK4a) functional peptide	[40,49,77]
P28	LSTAADMQGVVTDGMASGLDKDYLKPDD	p28, an anionic cell-penetrating peptide, increases the activity of wild type and mutated p53.	MCF-7, MDA-MB-231, and T47D, HCT116 and HT29, HT1080, (HTB-88), osteosarcoma (TE85), rhabdomyosarcoma (RD), glioblastoma (U87 and LN229), neuroblastoma (SK-N-BE2), prostate cancer (DU145), pancreatic cancer (MIA-Paca2) and ovarian cancer (ES-2)]. Melanoma lines (UISO-Mel-23, 29	P28	[78]
RALA peptide	WEARLARALARALARHLARALARA	Readily traversed the plasma membrane of both cancer and fibroblast cell lines and elicited reporter-gene expression following intravenous delivery in vivo*.*	ZR-75-1 human breast cancer, PC-3 human prostate cancerand NCTC-929 murine fibroblast cell lines	Plasmid DNA	[79]
TAT(47–57) Penetratin PEP-1 DS4.3	YGRKKRRQRRR RQIKIWFQNRRMKWKK KETWWETWWTEWSQPKKKRKV RIMRILRILKLAR	Anti-tumoral effect of the mitochondrial target domain of Noxa delivered by an engineered Salmonella typhimurium.	Male Balb/c mice; CT26 mouse colon cancer cells, HeLa and Hep3B cells	Mitochondrial Target Domain of NOXA	[80]
SR9 HR9 PR9	SRRRRRRRRR CHHHHHRRRRRRRRRHHHHHC FFLIPKGRRRRRRRRR	Direct membrane traslocation. Enhance the gene expression intensity.	A549 cells	Plasmid DNA	[81]
PF14	AGYLLGKLLOOLAAAALOOLL	Delivery pDNA forming stable nanoparticles that improve the transfection efficiency.	HeLa pLuc705 cells	Nucleic acids	[82,83]
d-NTD q-NTD	KGRKKRRQRRRPPQ KGRKKRRQRRRPPQ	d-NTD is the most potent conjugate against HepG2 human liver cancer cells.	HepG2	Doxorubicin	[84]
TH	AGYLLGHINLHHLAHL(Aib)HHIL-NH2	Acid-activated pH response for targeting delivery of antitumor drugs.	Hela cells	pH-responsive	[85]

* Amino acids in capital letters are in l configuration while those in lower case letters are in d configuration; Fl = Fluorescein moiety; Ahx = aminohexanoic acid; O = ornithine; Aib = aminoisobutyric acid.
